# Artificial sodium-selective ionic device based on crown-ether crystals with subnanometer pores

**DOI:** 10.1038/s41467-021-25597-1

**Published:** 2021-09-01

**Authors:** Tingyan Ye, Gaolei Hou, Wen Li, Chaofeng Wang, Kangyan Yi, Nannan Liu, Jian Liu, Shaoming Huang, Jun Gao

**Affiliations:** 1 Key Laboratory of Carbon Materials of Zhejiang Province, College of Chemistry & Materials Engineering, Wenzhou University, Wenzhou, China; 2 KU Leuven, Quantum Solid-State Physics section, Department of Physics and Astronomy, Leuven, Belgium; 3 College of Material Science and Engineering, Qingdao University of Science and Technology, Qingdao, China; 4 School of Materials and Energy, Guangzhou Key Laboratory of Low-Dimensional Materials and Energy Storage Devices, Guangdong University of Technology, Guangzhou, China; 5 Qingdao Institute of Bioenergy and Bioprocess Technology, Chinese Academy of Sciences, Qingdao, China; 6 Haiyu Chemical Engineering Co. Ltd, Dongying, China

**Keywords:** Sensors and biosensors, Nanosensors, Characterization and analytical techniques

## Abstract

Biological sodium channels ferry sodium ions across the lipid membrane while rejecting potassium ions and other metal ions. Realizing such ion selectivity in an artificial solid-state ionic device will enable new separation technologies but remains highly challenging. In this work, we report an artificial sodium-selective ionic device, built on synthesized porous crown-ether crystals which consist of densely packed 0.26-nm-wide pores. The Na^+^ selectivity of the artificial sodium-selective ionic device reached 15 against K^ + ^, which is comparable to the biological counterpart, 523 against Ca^2 + ^, which is nearly two orders of magnitude higher than the biological one, and 1128 against Mg^2 + ^. The selectivity may arise from the size effect and molecular recognition effect. This work may contribute to the understanding of the structure-performance relationship of ion selective nanopores.

## Abstract (web summary)

Artificial sodium channels open up the way to new separation technologies but remains highly challenging. In this work, the authors report an artificial sodium-selective ionic device, built on porous crown-ether crystals with a sodium ion selectivity against calcium ions exceeding that one of biological ion channel counterparts.

## Introduction

Biological ion channels, characterized by their smart ion transport property and distinguished ion selectivity, play a crucial role in many important biological processes^1,2^. For example, sodium channels have a Na^+^/K^+^ selectivity of ~10–10^2^ and a Na^+^/Ca^2+^ selectivity of ~7, serving to maintain the hemostasis equilibrium and transduce neural signals^3^. In the recent decades, great interest has been attracted to fabricate artificial ion channels, which can mimic some of the functions of the biological counterparts^4–11^. These artificial ion channels have shown promise for many important applications, such as molecular separation^12,13^, fit-for-purpose water treatment^4^, biosensor^14^, ionic circuitry^15–17^, energy conversion^18–22^, and lithium extraction^23,24^. However, while these artificial channels were able to control ion flow in various ways, for example, responsively to external stimuli^5,7^, they typically lacked the ability to distinguish different cations. This is because the simple cations present in the cellular environment (e.g., Na^+^, K^+^, Ca^2+^, Mg^2+^) have subnanometer sizes and their size differences range from <0.1 nm (bare K^+^ vs bare Na^+^) to ~0.2 nm (hydrated K^+^ vs hydrated Mg^2+^). These two factors pose extreme challenges to the structural engineering of the artificial ion channels: the pore size has to be small enough for ion discrimination and precise enough to accommodate only the target ion. In addition, ion transport rate in a nanopore generally follows the order of K^+^ > Na^+^. The opposite order Na^+^ > K^+^ was rarely found, posing another level of challenge to fabricate sodium ion channels.

Toward artificial ion channels, a conceptually straightforward method is to synthesize an organic architecture spanning through lipid bilayer membrane with ion transport activity, which mimics the configuration of biological ion channels^8–11^. However, the use of lipid bilayer renders the device fragile and less favorable in separation applications. Moreover, the Na^+^/K^+^ selectivity was either poor or absent^25^. Compared to synthetic artificial ion channels, solid-state artificial ionic devices consisting of nanochannels are much more robust and potentially more useful in applications^4–7^. To this end, metal-organic-frameworks, a highly porous material with a pore size precisely tunable from subnanometer to tens of nanometers^26^, were recently proposed to construct artificial ionic devices^27–30^. For example, by using a photosensitive organic linker, metal-organic-frameworks can be used as a photoresponsive ion channel or photodriven ion pump^29^. Remarkably, UiO-66-COOH metal-organic-frameworks with ~0.6 nm-sized windows and ~0.8–1.1 nm-sized cavities showed a high K^+^/Mg^2+^ selectivity of ~10^3^ and a Na^+^/Mg^2+^ selectivity of ~10^2^. However, the alkaline ion selectivity was only modest (K^+^/Na^+^ selectivity less than 2)^28^, and favors the transport of K^+^ over Na^+^. ZIF-8 metal-organic-frameworks, consisting of pores down to 0.3 nm, showed higher Na^+^ transport rate than K^+^, but the selectivity was still poor (slightly >1)^27^. Recently, Xin and co-workers further combined spatial confinement with charge-exclusion effects to enhance ion selectivity, and achieved a moderate Na^+^/K^+^ selectivity (less than 2)^24^. To the best of our knowledge, artificial sodium-selective ionic devices with a selectivity close to the biological counterparts have not been reported. Furthermore, the ability to simultaneously reject both K^+^ and other biogenic cations such as Ca^2+^ is also rarely realized in previous studies^25^.

A material frequently used to construct synthetic^9^ and sometimes solid-state^31,32^ ion channels is crown ether. Crown ether has a tunable ring that can bind specific ions, laying the foundation of ion selectivity. Furthermore, previous studies have shown that crown-ether-based porous crystals can be synthesized and these crystals can be used to transport ions^33–36^. It is therefore expected that if porous crown-ether crystals are rationally designed, it should be possible to realize artificial sodium-selective ionic device.

In this work, we report an artificial sodium-selective ionic device consisting of porous crown-ether crystals that favors the transport of Na^+^ over a variety of biogenic metal ions, i.e., K^+^, Ca^2+^, and Mg^2+^. In the crystal, the cavities of the crown ethers form tubular subnanometer pores. The narrowest part of the cavity, being 0.26 nm, is larger than the diameter of Na^+^ and slightly smaller than the diameter of K^+^. This type of structure should enable both high flux and high selectivity. The Na^+^/K^+^ selectivity, equaling 15, is comparable to the biological counterpart. Remarkably, the Na^+^/Ca^2+^ selectivity is 523, almost two orders of magnitude higher than the biological one.

## Results and discussion

### Material synthesis and device fabrication

**Fig. 1:**
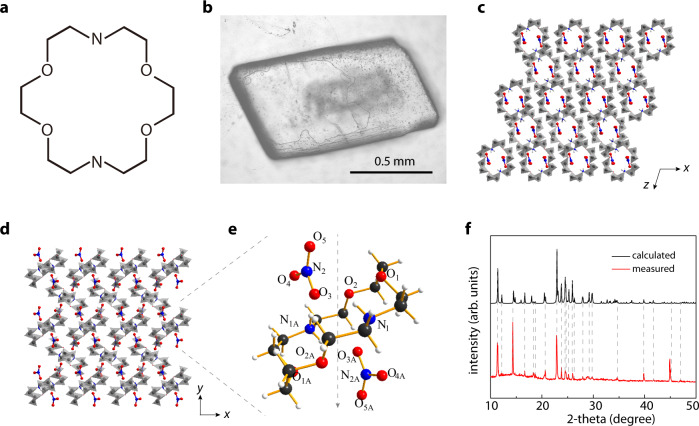
Synthesized crown-ether-based porous crystal. **a** Structure of the chosen crown ether, 1,10-diaza-18-crown-6-ether (DA18C6). **b** Picture of a synthesized DA18C6-nitrate single crystal. **c**, **d** Crystal structure viewed down [010] (**c**) and [001] (**d**) directions. In the [010] direction, the DA18C6 rings form densely packed subnanometer channels, allowing the transport of ions. The other two directions do not allow ion transport. **e** Magnified view of (**d**) and expected ion transport path (dashed line). Detailed crystal structure information can be found in the Supplementary information. Blue: nitrogen. Red: oxygen. Gray: carbon. **f** To verify the structure, we calculated theoretical powder X-ray diffraction pattern of the crystal, and measured the experimental one, whose results are in good agreement.

The crown ether used in this study is 1,10-diaza-18-crown-6-ether (DA18C6, molecular formula: C_12_H_26_N_2_O_4_. Fig 1a), which can bind Na^+^ strongly^37^. The narrowest part of the cavity is ~0.26 nm, which is larger than the diameter of Na^+^ (0.20 nm) and slightly smaller than the diameter of K^+^ (0.27 nm). In the “free” form, DA18C6 is conformationally flexible^38^, and can accommodate ions larger than the cavity. This would potentially affect its ion selectivity. We therefore aimed to improve the rigidity by assembling them into a porous crystal. If the pore is rigid enough, the transport of K^+^ could be efficiently suppressed even though it is only slightly smaller than the pore size. To this end, DA18C6 was dissolved in methanol, and mixed with methanol solution of Zn(NO_3_)_2_·6H_2_O at 70 °C. Then the mixture was refluxed, followed by cooling at 4 °C for sufficiently long time to produce white crystals (Fig. 1b and Supplementary Fig. 1, see experimental details in the Methods section). These crystals typically had a size of hundreds of micrometers to 1 mm. Such a large size allows us to easily solve the detailed crystalline structure, including the unit cell dimension, bond length, bond angle, and site ordering, using single-crystal X-ray diffraction. The resolved structure information is presented in Supplementary Table 1–2 and Supplementary Fig. 2. Indexing and refinement gave a monoclinic crystal (a = 779.02(16) pm, α = 90°, b = 1062.9(2) pm, β = 106.454(4)°, c = 1181.2(2) pm, γ = 90°) of DA18C6-nitrate ([C_12_H_28_N_2_O_4_](NO_3_)_2_), with a P21/n space group. The structures viewed down [010], [100], and [001] directions are presented in Fig. 1c, Supplementary Fig. 3, and Fig. 1d, respectively. In the [010] direction, the DA18C6 rings form parallel channels, allowing ion transport (expected ion transport path illustrated in Fig. 1e), as also verified with energy-dispersive X-ray characterizations (Supplementary Fig. 4). In the other two directions, there is not enough space for ion transport.

To further confirm the structure, we simulated the powder X-ray diffraction pattern of the solved structure and compared the pattern with the measured one. The results show good agreement (Fig. 1f). The relative intensities of the peaks are not exactly the same, probably because our submillimeter-sized crystals not really “powder”. In addition, to confirm the existence of –NO_3_ in the crystal, we characterized the Raman spectra of the crystal and DA18C6 (Supplementary Fig. 5). The crystal showed an extra strong peak at 1041 cm^−1^, which indeed can be assigned to –NO_3_. The –NO_3_ group is coordinated to the protonated DA18C6 ring.

**Fig. 2:**
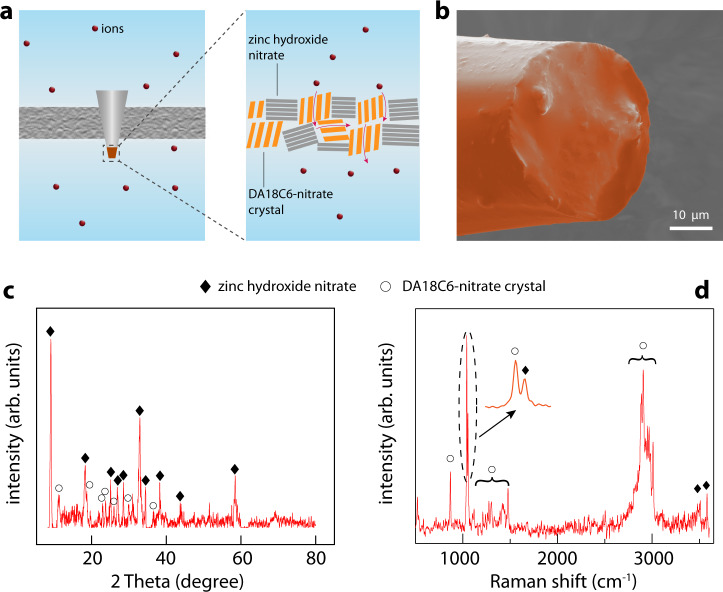
Preparation of the artificial sodium-selective ionic device. **a** The tip of a micropipette was filled with porous DA18C6-nitrate crystals which sieves ions, and nonporous zinc hydroxide nitrate which mechanically confines the DA18C6-nitrate crystals. The micropipette was placed between two electrolyte reservoirs to measure the ion transport property. **b** Scanning electron microscope image of the filled micropipette tip. **c** X-ray diffraction pattern of the filling mixture. Major peaks can be assigned to either DA18C6-nitrate crystal or zinc hydroxide nitrate (see also Fig. 1f and Supplementary Fig. 5). **d** Raman spectrum of the mixture, whose peaks can also be assigned to either DA18C6-nitrate crystal or zinc hydroxide nitrate (see also Supplementary Figs. 3 and 6). These results confirm that the filling mixture is composed of DA18C6-nitrate crystals and zinc hydroxide nitrate.

It is worth noting that large single crystals are not favored for practical applications due to their poor processability. When Zn(NO_3_)_2_·6H_2_O and DA18C6 react at room temperature, the growth speed of crystal should be slow, and may produce small crystals with higher processability. We therefore filled the tip of a quartz micropipette (inner diameter: 23 ± 3 μm) with Zn(NO_3_)_2_·6H_2_O and DA18C6 solutions at room temperature (Methods and Supplementary Fig. 6). Zn(NO_3_)_2_·6H_2_O transfers a part of the –NO_3_ groups and protons to DA18C6 to form the DA18C6-nitrate crystals, and the other product is thus presumably zinc hydroxide nitrate, a nonporous layered compound^39^, functioning as a matrix to support the DA18C6-nitrate crystals (Fig. 2a–c).

$$	5\;{{{{{\rm{Zn}}}}}}{({{{{{{\rm{NO}}}}}}}_{3})}_{2}+4\;{{{{{\rm{C}}}}}}_{12}{{{{{{\rm{H}}}}}}}_{26}{{{{{{\rm{N}}}}}}}_{2}{{{{{{\rm{O}}}}}}}_{4}+8\;{{{{{\rm{H}}}}}}_{2}{{{{{\rm{O}}}}}}\\ 	\;\;\;\to {{{{{{\rm{Zn}}}}}}}_{5}{({{{{{\rm{OH}}}}}})}_{8}{({{{{{{\rm{NO}}}}}}}_{3})}_{2}+ 4\;[{{{{{{\rm{C}}}}}}}_{12}{{{{{{\rm{H}}}}}}}_{28}{{{{{{\rm{N}}}}}}}_{2}{{{{{{\rm{O}}}}}}}_{4}]{({{{{{{\rm{NO}}}}}}}_{3})}_{2}$$

After the tip was filled, we can conveniently measure the ion transport property by placing the micropipette between two electrolyte reservoirs (Fig. 2a–b). The scanning electron microscope image (Fig. 2b) shows that the DA18C6-nitrate/zinc hydroxide nitrate mixture is largely homogeneous and completely sealed the micropipette tip without observable pinholes. Therefore, we expect a large part of the ions to transport through the DA18C6-nitrate crystals. To confirm that the mixture is composed of zinc nitrate hydroxide and DA18C6-nitrate crystals, we first examined the powder X-ray diffraction (XRD) pattern of the mixture. Almost all XRD peaks of the mixture can be assigned either to zinc nitrate hydroxide (separately synthesized for control measurements, see Methods) or DA18C6-nitrate crystal (Fig. 2c and Supplementary Fig. 7). In addition, we collected the confocal Raman spectra. The bands of the mixture contained only those of zinc nitrate hydroxide and those of DA18C6-nitrate (Fig. 2d and Supplementary Fig. 8).

Once the device is ready, we first checked the role of the zinc hydroxide nitrate. We increased the molar fraction of zinc hydroxide nitrate from 20% (i.e., the original fraction described above) to 100%, and found that the ionic conductivity decreased by 4 orders of magnitude (Supplementary Fig. 9). This result confirms that zinc hydroxide nitrate does not conduct ions, functioning as a matrix as expected. Note that if no zinc hydroxide nitrate is used, the DA18C6-nitrate cannot be self-supporting (Supplementary Fig. 10).

### Ion selectivity characterization

**Fig. 3:**
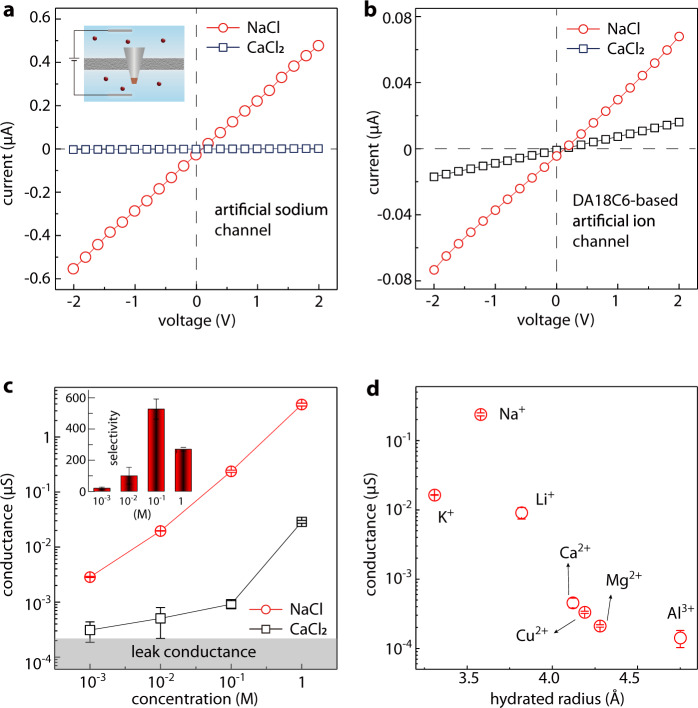
Na^+^/Ca^2+^selectivity of the artificial sodium-selective ionic device. **a***I–V* curves for 0.1 M NaCl and 0.1 M CaCl_2_ transporting through the artificial sodium-selective ionic device. The Na^+^/Ca^2+^ selectivity was calculated to be 523. Inset: schematic illustration of the setup. **b** To verify the important role of the porous crystal structure of the DA18C6-nitrate, we fabricated a noncrystalline-DA18C6-based artificial ion channel, which displayed a dramatically lower selectivity of ~8. **c** The conductance of the NaCl and CaCl_2_ through the artificial sodium-selective ionic device as a function of concentration. Inset: calculated selectivity, showing a peak at 0.1 M. **d** The conductance of various 0.1 M chlorides, showing that the conductance generally decreases with the increase of hydrated radium, suggesting the importance of size selectivity. Error bars in all cases indicate the standard deviation of the data.

Toward ion selectivity characterization, we then filled the electrolyte reservoirs with 0.1 M LiCl, NaCl, KCl, CuCl_2_, CaCl_2_, MgCl_2_, or AlCl_3_, and tested their conductivities, respectively. It is expected that the solution pH value affects the ionic current (Supplementary Fig. 11). For monovalent salts, the ionic current reached maximum when the solution was nearly neutral. For simplicity, in our ion selectivity characterization experiments, we used freshly prepared solutions without tuning pH. Typical current–voltage (*I–V*) curves for NaCl and CaCl_2_ are displayed in Fig. 3a. While Na^+^ exhibited a high conductance of ~0.24 μS, Ca^2+^ only had a conductance of ~9.17 × 10^−4^ μS. The Na^+^/Ca^2+^ selectivity is calculated to be,

1 $${{{{{\rm{Selectivity}}}}}}=\frac{{G}_{{{{{{\mathrm{Na}}}}}}}}{{G}_{{{{{{\mathrm{Ca}}}}}}}}\cdot \frac{{z}_{{{{{{\mathrm{Ca}}}}}}}}{{z}_{{{{{{\mathrm{Na}}}}}}}}=523,$$

where *G* is the conductance, and *z* is the valence number for Na^+^ or Ca^2+^. This Na^+^/Ca^2+^ selectivity far exceeds previous studies^28,40^ and is two orders of magnitude higher than the biological ion channels^3^. To elucidate the role of porous structure of the DA18C6-nitrate crystal, we fabricated an artificial ion channel by filling the micropipette tip with a mixture of DA18C6 and zinc hydroxide nitrate (denoted as DA18C6-based artificial ion channel). The DA18C6 still imparted the device with Na^+^/Ca^2+^ selectivity. However, without the ordered porous structure, the selectivity was dramatically lower (~8, *I–V* curves shown in Fig. 3b). In addition, we also measured Na^+^/Ca^2+^ selectivity of a blank micropipette, which is negligibly small (Supplementary Fig. 12).

For a more systematic characterization, we measured the conductance of NaCl and CaCl_2_ of different concentrations and summarized the selectivity (Fig. 3c). Below 0.1 M, it is observed that the selectivity decreases sharply with the decrease of concentration. This does not necessarily suggest the sharp decrease in performance. Instead, it could be attributed to the fact that in the low concentration region, the conductance of CaCl_2_ is comparable to the leak conductance (i.e., conductance of ultrapure water), and cannot accurately represent the Ca^2+^ transport. For the high concentration of 1 M, the selectivity is also lower than that in 0.1 M, probably due to the vanishing charge selectivity induced by the vanishing electric double layer^41^. Nevertheless, all these selectivities are higher than the biological sodium channels.

To understand the selectivity mechanism deeper, we recorded the conductance for the other electrolytes (Fig. 3d), which displayed a sequence of Na^+^ > Li^+^ >> Ca^2+^ >Cu^2+^ >Mg^2+^~Al^3+^. The selectivities of Na^+^/Li^+^, Na^+^/Cu^2+^, Na^+^/Mg^2+^, and Na^+^/Al^3+^ are calculated to be 26, 723, 1128, and 1681, respectively. This selectivity sequence shares the same order as the hydrated ionic radius (Fig. 3d), suggesting that the selectivity among these ions are governed by the ionic hydration energy. In fact, the hydration energy of all divalent and trivalent ions examined here are at least three times higher than that of Na^+^. The high Na^+^ selectivity was also kept in a multicomponent ion mixture (Supplementary Fig. 13). It should be noted that, to the best of our knowledge, all the above selectivity values are the highest ever reported. Although for biological sodium channels, the Na^+^/Li^+^, Na^+^/Cu^2+^, Na^+^/Mg^2+^, and Na^+^/Al^3+^ selectivities are of less importance due to the low concentration of these rival ions, they may be of great importance in some industrial separation applications where these ions are often present.

Besides metal ions, our device also allows the transport of protons, whose conductivity is slightly higher than Na^+^ (Supplementary Fig. 14).

### Na^+^/K^+^selectivity

**Fig. 4:**
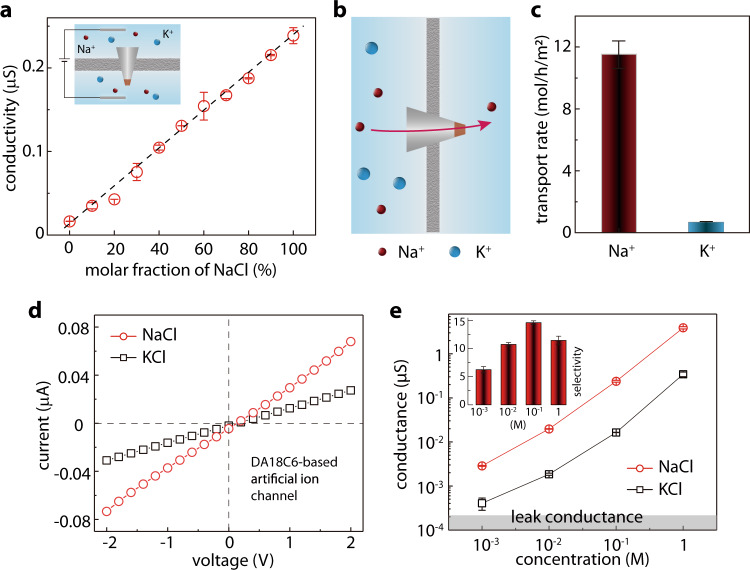
Na^+^/K^+^selectivity of the artificial sodium-selective ionic device. **a** Ionic conductance for NaCl + KCl mixture solutions with a total concentration of 0.1 M and variable NaCl molar ratios. The conductance increased almost linearly with the NaCl molar ratio, suggesting the reliable Na^+^/K^+^ selectivity in the binary ion mixture. Inset: schematic illustration of the setup. To further confirm this, we conducted ion diffusion measurement. **b** Schematic illustration of the diffusion experiment setup. The feed side reservoir (left) contained 0.1 M NaCl + 0.1 M KCl, and the permeate side (right) contained ultrapure water. **c** The measured transport rate of Na^+^ and K^+^. The calculated selectivity (~15) is also in agreement with the one extracted from ionic conductivity results. **d** To verify the important role of the porous crystal structure of the DA18C6-nitrate, we again tested the ion selectivity of the noncrystalline-DA18C6-based artificial ion channel, which is merely ~2. **e** The conductance of the NaCl and KCl through the artificial sodium-selective ionic device as a function of concentration. Inset: calculated selectivity. Error bars in all cases indicate the standard deviation of the data.

The selectivity of Na^+^ over K^+^ is important for the functions of biological sodium channels. Our artificial sodium-selective ionic device exhibited a high Na^+^/K^+^ selectivity of ~15 (Fig. 4c), comparable to the biological counterpart. In cyclic experiment, the selectivity kept at around 11.3–17.6 (Supplementary Fig. 15). At first sight, the higher Na^+^ conductance than K^+^ in Fig. 3b appears to be outlier. This suggests that the hydration energy may not play a dominant role here. Molecular recognition, i.e., the binding of the Na and the crown-ether macrocycle, may be more important. To examine this, we conducted density functional theory calculations (Methods). The binding energies of DA18C6-M (M = metal cation) are summarized in Supplementary Table 3. We found that the binding energy of DA18C6-Na^+^ (−385 kJ/mol) is more negative than DA18C6-K^+^ (−337 kJ/mol), meaning that DA18C6 favors Na^+^ binding. This molecular recognition may be responsible for the Na^+^/K^+^ selectivity of our device. Apparently, the binding energy (*E*_*binding*_) and the hydration energy (*E*_*hydration*_) play competing roles in the ion conductivity. A simplified model suggests that (see Supplementary Information),

2 $$G\propto {{{{{\mathrm{exp}}}}}}(|{E}_{{{{{{\mathrm{hydration}}}}}}}-{E}_{{{{{{\mathrm{binding}}}}}}}|)$$

The experimental results are indeed largely in agreement with this model (Supplementary Fig. 16). The discrepancies are expected because this simplified model does not account for the complex structure of composite material.

In living cells, the Na^+^/K^+^ competition is central to the neural signal transduction. Sieving out K^+^ from a mixture of Na^+^ and K^+^ is therefore of critical importance. The above results only recorded the conductance for either K^+^ or Na^+^. To test the selectivity in Na^+^/K^+^ mixture, we mixed the NaCl and KCl solutions with a total concentration of 0.1 M and variable Na^+^ molar ratio. Results show that the ionic current increased approximately linearly with the increase of Na^+^ molar ratio (Fig. 4a and Supplementary Fig. 17), suggesting that our device kept favoring the transport of Na^+^ regardless of the K^+^ presence. We further verify this result with an additional ion diffusion experiment (Fig. 4b, c). To do so, we mixed 0.1 M NaCl + 0.1 M KCl in the feed side reservoir and filled the permeate side with ultrapure water to allow the ion diffusion. After 48 h, we measured Na^+^ and K^+^ concentration in the permeate side with ion chromatography (results in Fig. 4c). In this way both the ion transport rate and ion selectivity can be quantified. The Na^+^/K^+^, equaling ~15, is in good agreement with the above results. Our artificial sodium-selective ionic device can be further extrapolated to a membrane, for example, by filtrating the mixture of zinc hydroxide nitrate and DA18C6-nitrate crystals on an anodic aluminum oxide film (Supplementary Fig. 18), showing a Na^+^/K^+^ selectivity of 3.6. The lower Na^+^ selectivity is attributed to the structural defects generated during filtration. Better membrane fabrication strategies are required in future studies.

For the Na^+^/K^+^ selectivity, the important role of the porous DA18C6-nitrate crystal was again supported by the negligible selectivity of a blank micropipette (Supplementary Fig. 18) and poor selectivity (~2) of the DA18C6-based artificial ion channel (Fig. 4d). Similar to Na^+^/Ca^2+^, the Na^+^/K^+^ selectivity also increased gradually when the ion concentration increased from 1 mM to 0.1 M, and then decreased when the ion concentration increased to 1 M (Fig. 4e). The mechanism of this selectivity response should also be similar to that of the Na^+^/Ca^2+^ selectivity response in Fig. 3c. However, the decrease in the Na^+^/K^+^ selectivity in the low concentration region is much slower than the Na^+^/Ca^2+^ selectivity, thanks to the significantly higher K^+^ current than the leak conductance. Even the lowest Na^+^/K^+^ selectivity (~6) is still higher than previous studies^25,27^, and is not dramatically lower than the biological sodium channels.

In summary, we have developed an artificial sodium-selective ionic device with high selectivity against a variety of biogenic metal ions. The Na^+^/K^+^ selectivity up to 15, is comparable to the biological sodium channels and the Na^+^/Ca^2+^ selectivity is up to 523, two orders of magnitude higher than the biological one. The high selectivity should be attributed to the successful fabrication of porous crown crystals, which consist of dense pores with a small size of ~0.26 nm. These narrow pores block the transport of most hydrated rival ions but favors the transport of Na^+^, which has a small enough bare size and has molecular recognition interaction with the crown-ether macrocycle. Since crown ethers are a class of materials with variable cavity size and functional groups, it should be possible to construct various artificial ion channels with tunable ion selectivity, which would open more possibilities in separation and biosensing technologies.

## Methods

### Synthesis of DA18C6-nitrate crystals

Zn(NO_3_)_2_·6H_2_O (0.446 g, 1.5 mmol) was dissolved in hot methanol (10 mL), followed by adding 7.5 mL 1.5 mM methanol solution of 1,10‐diaza‐18‐crown‐6. The mixed solution was refluxed for 3–4 days at 70 ± 2 °C until the solution volume decreased to ~5 mL. Then the solution was sealed and kept still at 4 °C for 2 days to allow the growth of crystals (Supplementary Fig. 1).

### Preparation of artificial sodium-selective ionic device

Quartz micropipette was fabricated by pulling quartz capillaries with Sutter P-97. The tip diameter of the micropipette was typically a few tens of micrometers. To prepare artificial sodium-selective ionic device, 104.94 mg 1,10‐diaza‐18‐crown‐6 was dissolved in 5 mL of dichloromethane and 5 mL of methanol at 25 °C. Then 2.2 ± 0.2 μL such solution was added into the prepared micropipette. After that, the micropipette was immersed in 10 mL methanol solution of 0.625 mmol Zn(NO_3_)_2_·6H_2_O for 20 sec. Finally, we kept the micropipette hanging vertically in air at room temperature for 8 h. A mixture of zinc hydroxide (Zn_5_(OH)_8_(NO_3_)_2_) and DA18C6-nitrate crystal filled the tip of the micropipette. The thickness of the mixture was about 25 μm.

### Preparation of the DA18C6-based artificial ion channel

77.87 mg Zn_5_(OH)_8_(NO_3_)_2_·2H_2_O and 104.94 mg DA18C6 were dissolved in a mixture of 5 mL methanol and 5 mL dichloromethane, followed by sonicating for 3 h until a uniform suspension formed. 4.0 ± 0.4 μL suspension was added into the micropipette. Finally, we kept the micropipette hanging vertically in air at room temperature for 8 h.

### Synthesis of zinc hydroxide nitrate (Zn_5_(OH)_8_(NO_3_)_2_·2H_2_O)

Aqueous NaOH solution (400 mg NaOH in 50 mL water) was slowly added to Zn(NO_3_)_2_·6H_2_O solution (2.97 g Zn(NO_3_)_2_·6H_2_O in 50 mL water) under stirring. After stirring for 30 min, white precipitate was observed, which was washed at least three times with abundant ultrapure water under vacuum filtration. After washing, the powder was dried at 50 °C for 24 h.

### Material characterization

The crystal was analyzed with single-crystal X-ray diffraction (Bruker-AXS SMART APE II DUO diffractometer) at 296(2) K. The crystal structure was solved and refined following routine procedures with H atoms of the crowns added to the calculated positions. More details can be found in Supplementary Table 1–2. Powder X-ray diffraction was completed on Bruker D8 ADVANCE. Scanning electron microscope (SEM) was carried out with Nova 200 NanoSEM and Raman measurements were carried out with Renishaw Invia laser microraman spectrometer.

### Ion selectivity characterization

The prepared artificial sodium-selective ionic device and the DA18C6-based artificial ion channel were placed between two electrolyte reservoirs made of polytetrafluoroethylene (Fig. 3a in the main manuscript). The reservoirs were thoroughly rinsed with ultrapure water before testing. To test the ion selectivity, we first characterized the ionic current response under sweeping voltages with Keithley 6487 sourcemeter for various electrolyte type and concentration. From the measured ionic current and applied voltage, we derive the ionic conductance. After filling electrolyte and before electrical measurement, the device was kept still for tens of minutes to reach equilibrium. The conductivities of various electrolyte were compared to analyze the selectivity. Separation of binary ion mixture was analyzed with ion chromatography. In short, we filled the feed side electrolyte with 0.1 M NaCl+0.1 M KCl, and filled the permeate side with ultrapure water. Then the device was kept still for 48 h, after which the Na^+^ and K^+^ concentration were measured with ion chromatography (Metrohm 930 Compact IC Flex) to derive the ion transport rate and ion selectivity.

### Theoretical calculations

To better understand ion selectivity, we conducted density functional theory (DFT) calculations on the simplified model complexes, i.e., DA18C6-M (M = Na^+^, K^+^, Ca^2+^, Mg^2+^, Cu^2+^, and Al^3+^). The geometric structures were optimized using the B3LYP functional^42,43^ with Grimme’s dispersion corrections (D3BJ)^44^. The Def2-TZVP^45^ basis set was employed for all atoms. Harmonic frequency calculations have been carried out to verify that all the structures are true minima. The binding energies between DA18C6 and metal ions were calculated including zero-point vibrational energy corrections. All the calculations were performed with Gaussian09 software package^46^.

## Supplementary information

Supplementary Information

## Data Availability

The experiment data that support the findings of this work are available from the corresponding authors upon reasonable request. The X-ray crystallographic coordinates for structures reported in this study have been deposited at the Cambridge Crystallographic Data Centre (CCDC), under deposition numbers 2055887. These data can be obtained free of charge from The Cambridge Crystallographic Data Centre via www.ccdc.cam.ac.uk/data_request/cif.
