# Supplementary material for: Artificial sodium-selective ionic device based on crown-ether crystals with subnanometer pores
Source: Nat Commun. 2021 Sep 1;12:5231. doi: 10.1038/s41467-021-25597-1 (PMC8410819; doi:10.1038/s41467-021-25597-1)
Supplement: Supplementary file 1 — Supplementary Information [file 41467_2021_25597_MOESM1_ESM.pdf]

Supplementary Information for

Artificial sodium-selective ionic device based on crown-ether crystals with subnanometer pores

Tingyan Ye[1], Gaolei Hou[2], Wen Li[1], Chaofeng Wang[1], Kangyan Yi[1], Nannan Liu[1, *], Jian Liu[3], Shaoming Huang[4, *], Jun Gao[5, 6,*]

[1] Key Laboratory of Carbon Materials of Zhejiang Province, College of Chemistry & Materials Engineering, Wenzhou University, Wenzhou 325027, China

[2] KU Leuven, Quantum Solid-State Physics section, Department of Physics and Astronomy, Celestijnenlaan 200D, Leuven 3001, Belgium

[3] College of Material Science and Engineering, Qingdao University of Science and Technology, Qingdao 266042, China

[4] School of Materials and Energy, Guangzhou Key Laboratory of Low-Dimensional Materials and Energy Storage Devices, Guangdong University of Technology, Guangzhou 510006, China.

[5] Qingdao Institute of Bioenergy and Bioprocess Technology, Chinese Academy of Sciences, Qingdao, 266101 China

[6] Haiyu Chemical Engineering Co. Ltd, Dongying 257506, China

[*] Email: liunannan@wzu.edu.cn; smhuang@gdut.edu.cn; jun.gao@qibebt.ac.cn

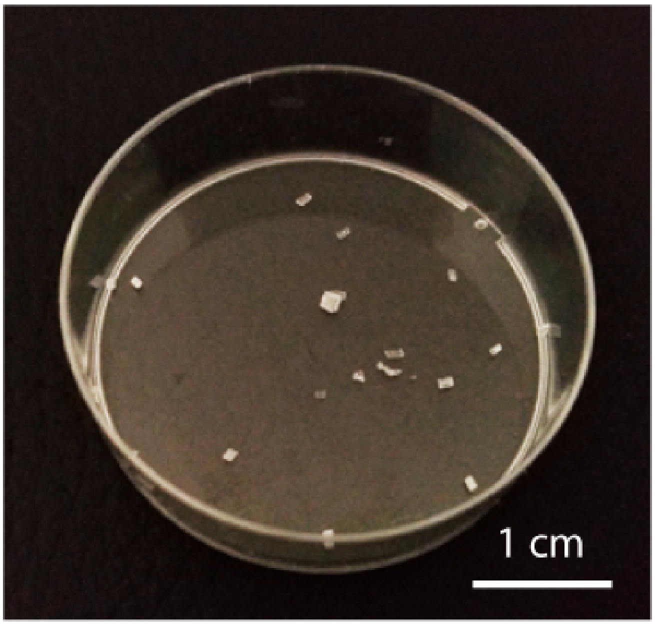

Supplementary Figure 1 | Picture of the DA18C6-nitrate crystals in a Petri dish.

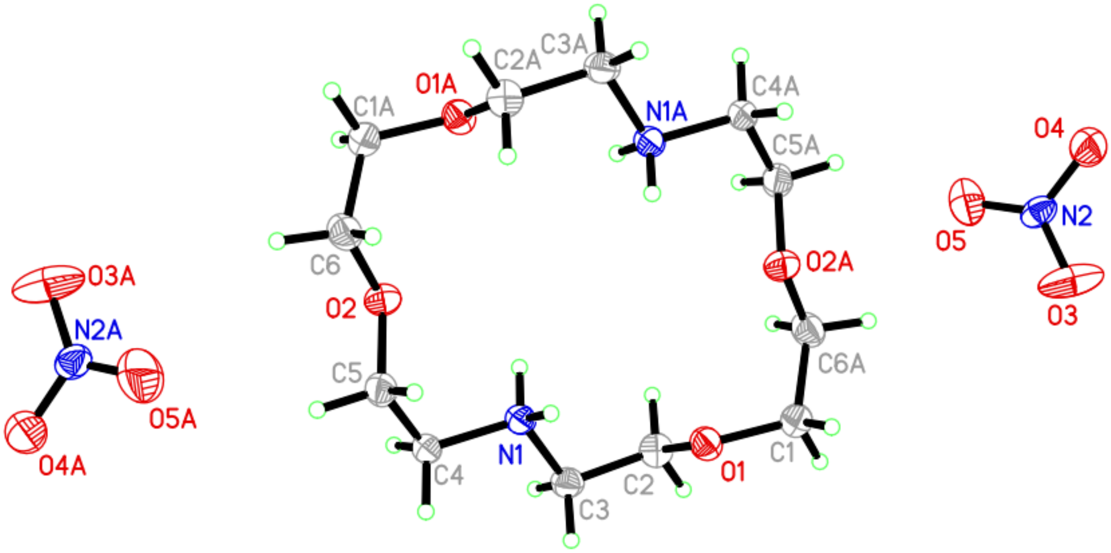

Supplementary Figure 2 | Crystal unit of the DA18C6-nitrate. Detailed crystal structure can be found in Supplementary Table 1 and 2.

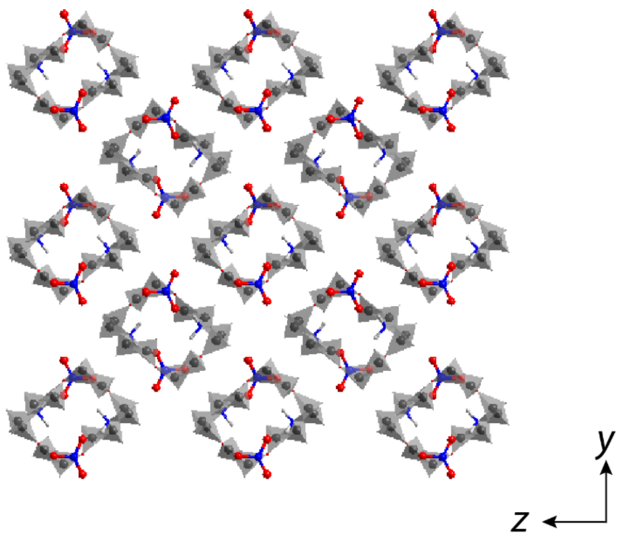

Supplementary Figure 3 | Crystal structure viewed down [100] direction.

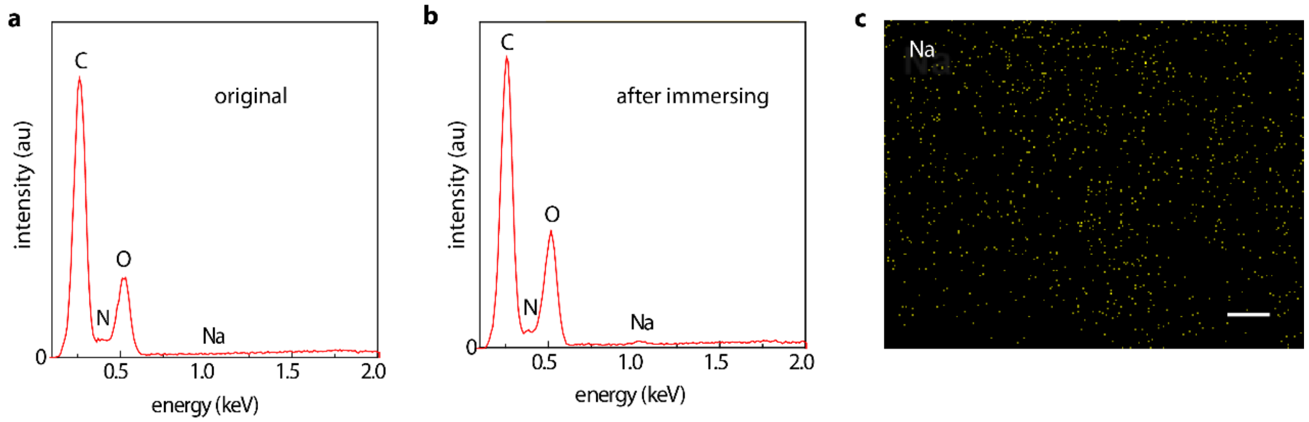

Supplementary Figure 4 | Na permeated in a crown ether crystal. A single crown ether crystal was immersed in 1.71×10^{-3} M NaCl solution for 72 h. Then it was mechanically cleaved to expose a fresh inner surface for Energy-dispersive X-ray spectroscopy (EDX) characterization. (a) EDX spectrum before immersing. (b) EDX spectrum after immersing. (c) EDX mapping of the Na element after immersing. Scale bar: 25 μm.

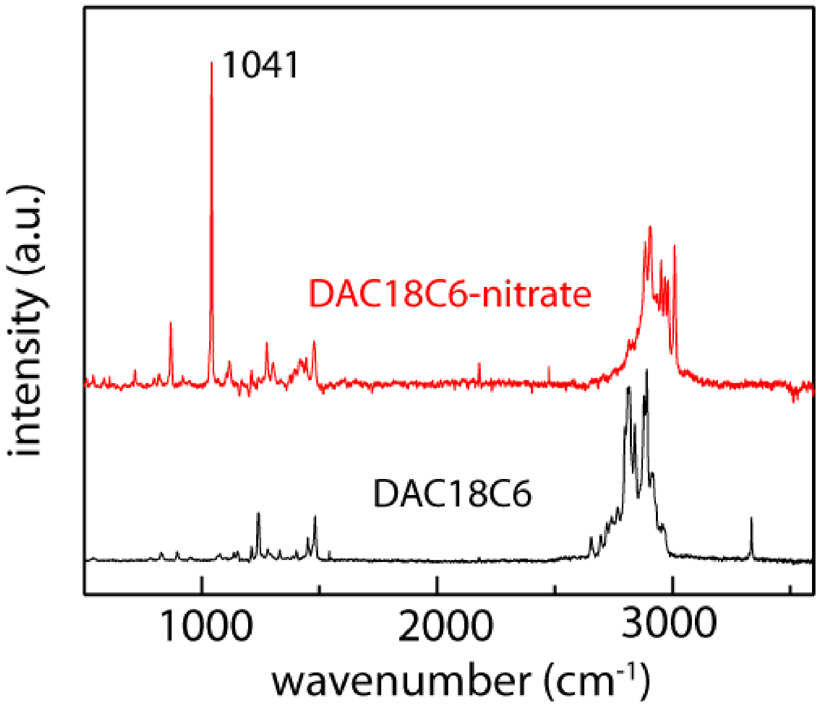

Supplementary Figure 5 | Raman spectra of DA18C6 and DA18C6-nitrate. Compared to DA18C6, DA18C6-nitrate has an extra strong peak at 1041 cm^{-1}, which can be assigned to $-NO_3$. In addition, the broad bands right below 3000 cm^{-1}, which can be assigned to C-H stretching on the crown ether ring, shifted location, indicating that the crown ether ring is coordinated with functional groups.

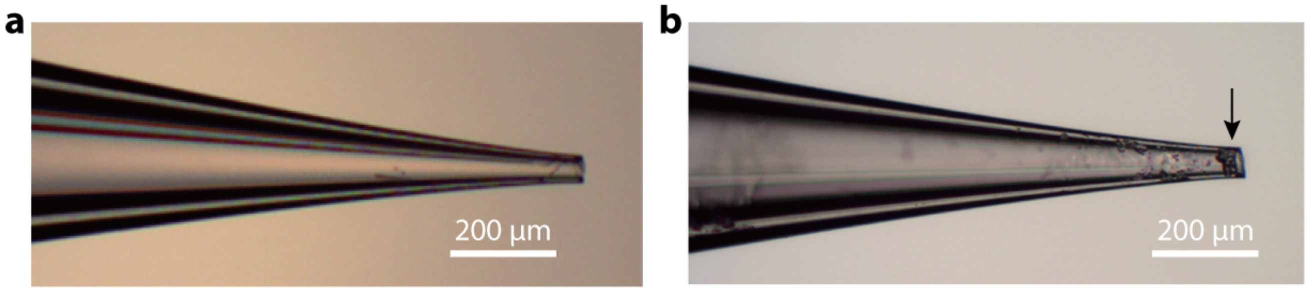

Supplementary Figure 6 | Picture of a quartz micropipette (a) and artificial sodium channel prepared with the micropipette (b). The DA18C6-nitrate crystals and zinc hydroxide nitrate filled the tip of the micropipette.

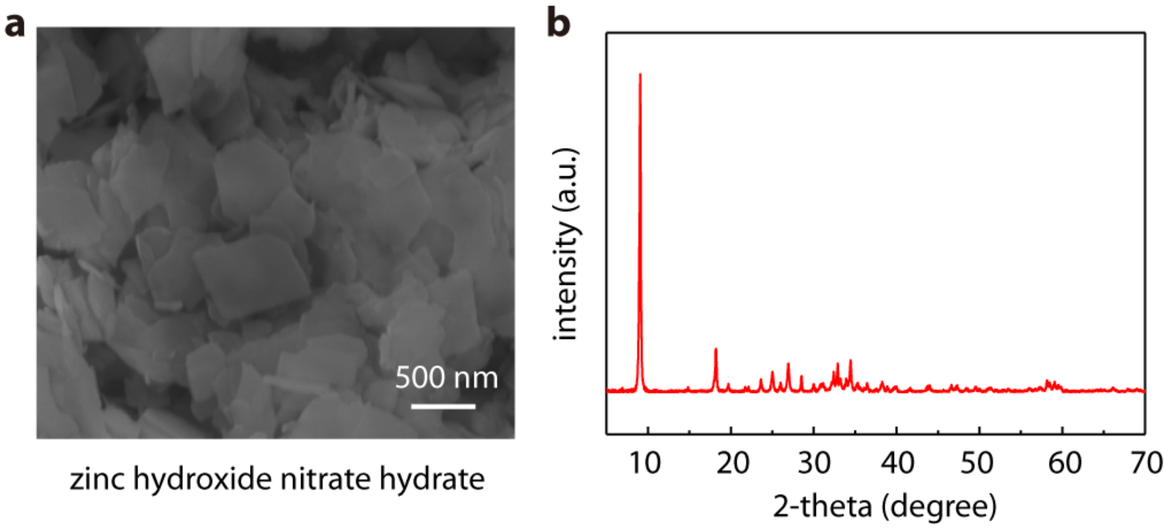

Supplementary Figure 7 | SEM image of the zinc hydroxide nitrate (a) and the powder X-ray diffraction pattern (b).

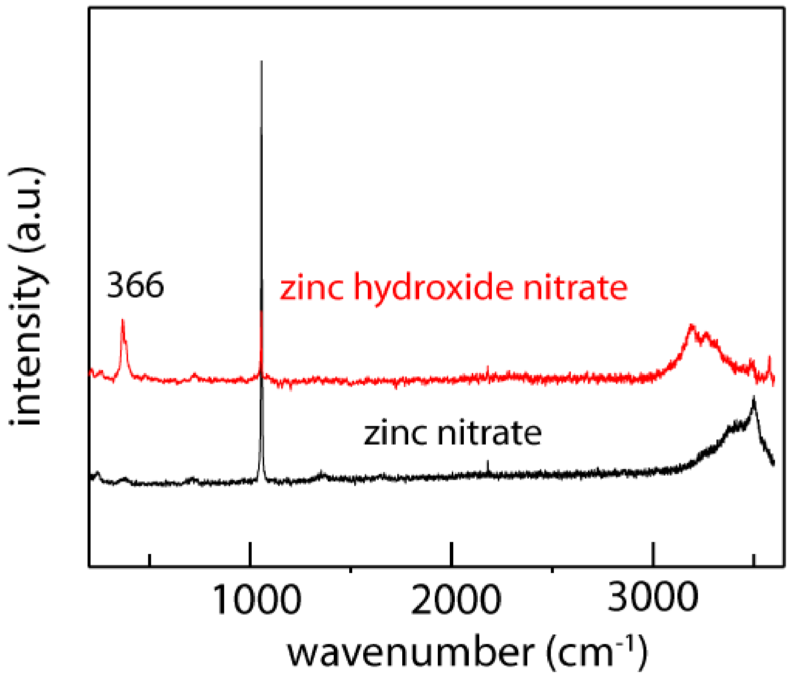

Supplementary Figure 8 | Raman spectra of zinc nitrate and zinc hydroxide nitrate. Compared to zinc nitrate, zinc hydroxide nitrate has an extra strong peak at 366 cm^{-1}, which can be assigned to Zn-OH. In addition, zinc hydroxide nitrate has more peaks between 3000-3650 cm^{-1}, indicating the existence of additional OH groups since this region can be assigned to various OH stretching modes.

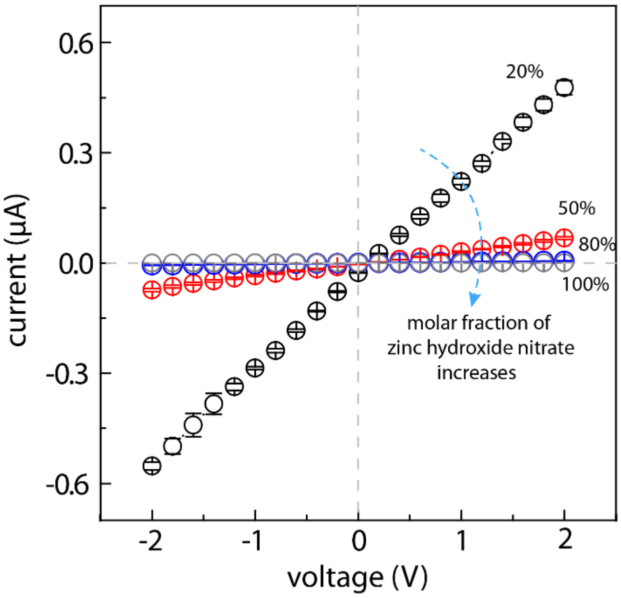

Supplementary Figure 9 | *I-V* response to the molar fraction of zinc hydroxide nitrate. We increased the molar fraction of zinc hydroxide nitrate from 20% (*i.e.*, the original fraction) to 100%, and found that the ionic conductivity decreased dramatically. This result confirms that zinc hydroxide nitrate does not conduct ions, functioning as a matrix as expected. Error bars in all cases indicate the standard deviation of the data.

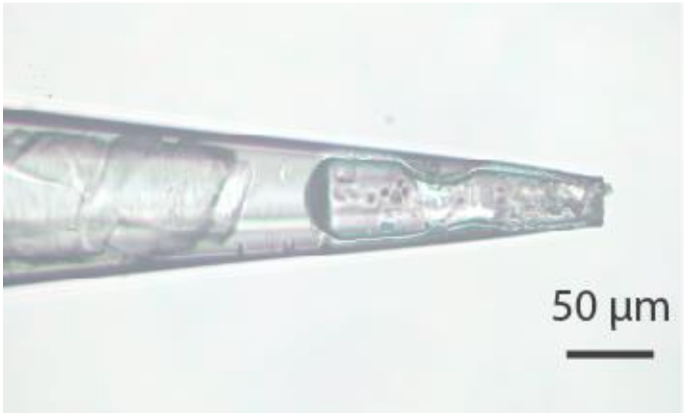

Supplementary Figure 10 | Optical image of a quartz micropipette modified only with porous crown-ether crystals after immersed in water for 1 minute. The crystals could not seal the micropipette and therefore water could be seen flowing through.

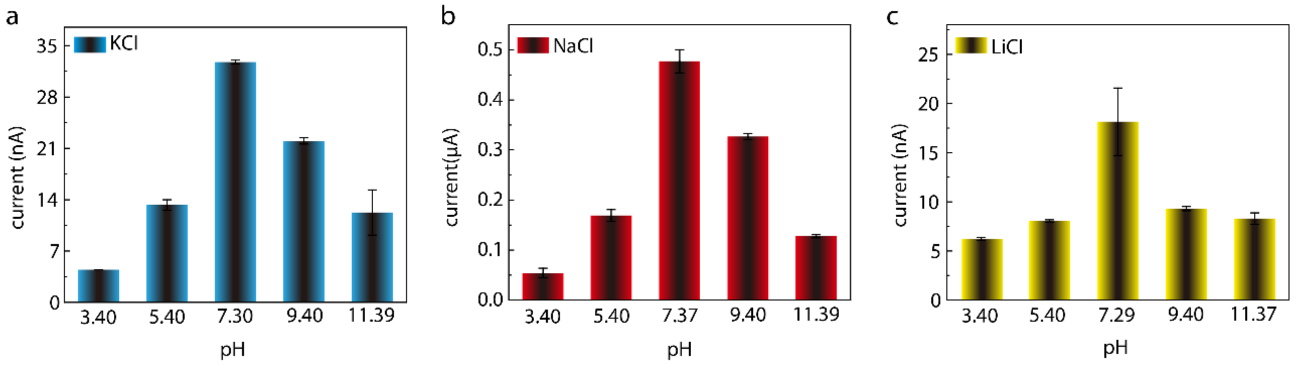

Supplementary Figure 11 | Response of ionic current to pH for different electrolytes at 2 V. (a) 0.1 M KCl. (b) 0.1 M NaCl. (c) 0.1 M LiCl. Error bars in all cases indicate the standard deviation of the data.

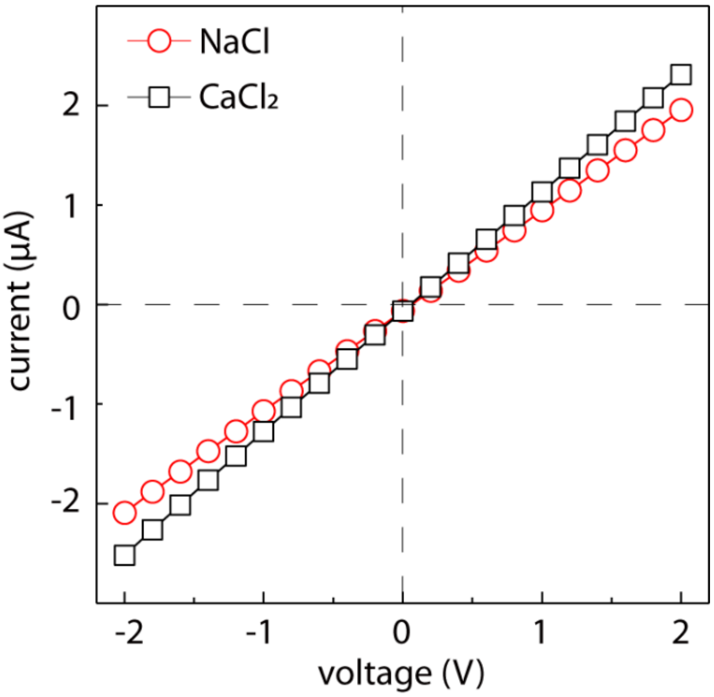

Supplementary Figure 12 | *I-V* curves of 0.1 M NaCl and 0.1 M $CaCl_2$ with blank micropipette, showing negligible selectivity.

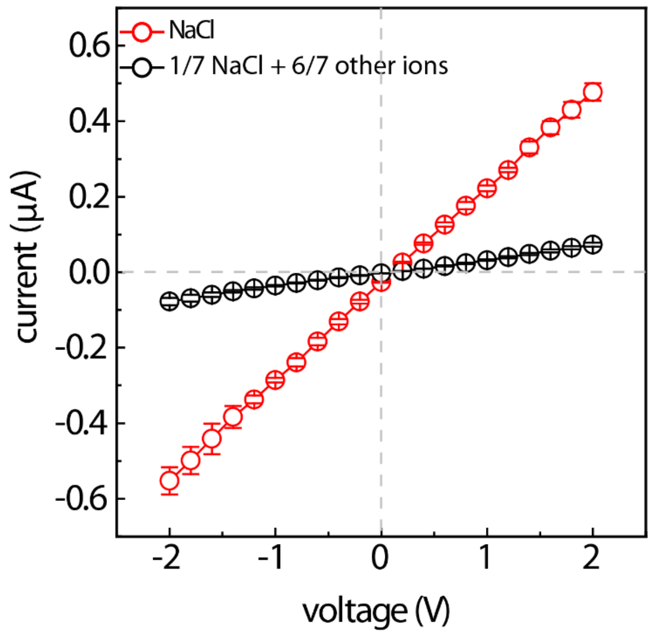

Supplementary Figure 13 | Na^+ selectivity in mixed ions tested with *I-V* curves. NaCl, KCl, LiCl, $CuCl_2$, $CaCl_2$, $MgCl_2$ and $AlCl_3$ solutions with the same concentration (0.1 M) were mixed. The resultant solution has a total molar concentration of 0.1 M, and NaCl has a molar fraction ratio of 1/7. If our device keeps high Na^+ selectivity, the ionic current would be 1/7 of 0.1 M NaCl. The result was indeed in agreement with this deduction. Error bars in all cases indicate the standard deviation of the data.

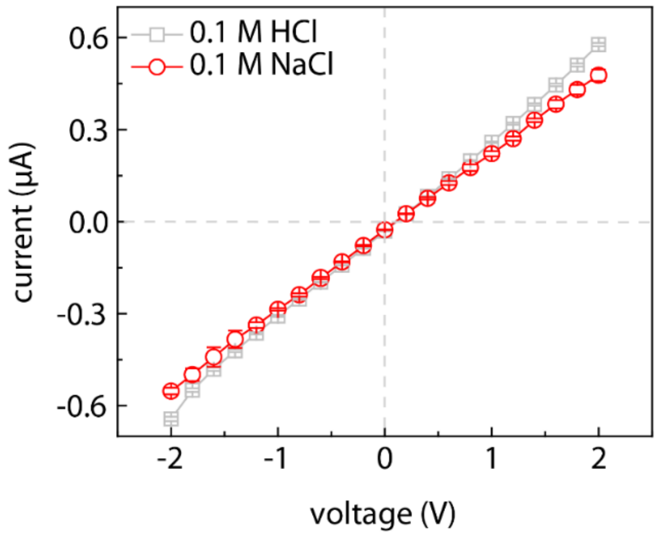

Supplementary Figure 14 | *I-V* curves for 0.1 M HCl and 0.1 M NaCl. The result showed that proton transports slightly faster than Na^+ in our device. Error bars in all cases indicate the standard deviation of the data.

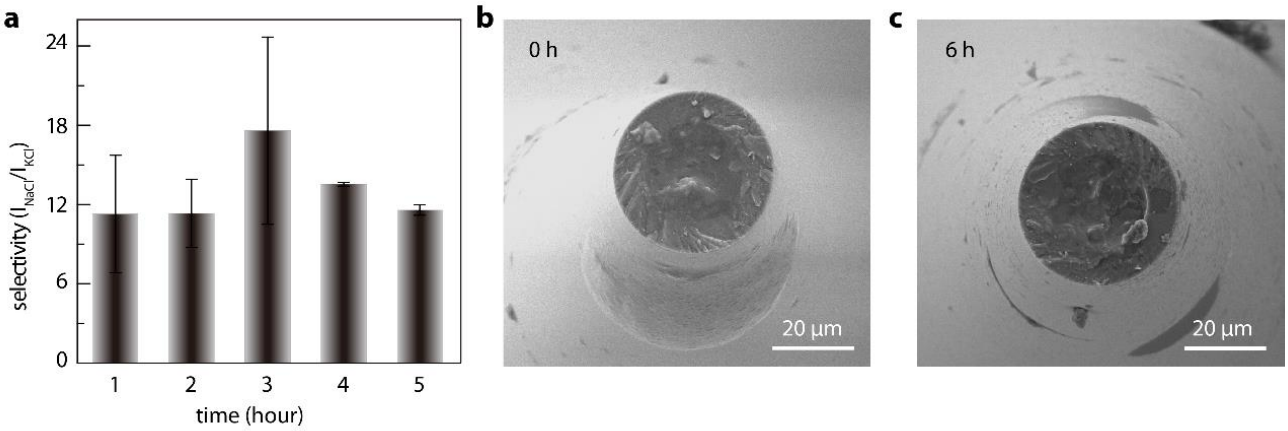

Supplementary Figure 15 |Repeatable high Na^+/K^+ selectivity in cyclic experiment. (a) The device were immersed in testing solution for 6 hours and the Na^+/K^+ were tested, showing that the selectivity kept at a high value. SEM images before testing (b) and after testing (c) show that the device largely kept its original form. Error bars in all cases indicate the standard deviation of the data.

A model to analyze ion transport mechanism

To explore the transport mechanism and thereby the ion selectivity mechanism, we conducted density functional theory calculations (see Methods) to understand the interaction between the crown ether DA18C6 and the metal ions (Supplementary Table 3).

We found that Na^+ and DA18C6 has a binding energy of -385 kJ/mol (Supplementary Fig. 16), close to the hydration energy of Na^+ (-379 kJ/mol). This means that DA18C6 can well replace the hydration shell when Na^+ enters the macrocycles, significantly lower the energy barrier (ΔE) of ion transport. Here $\Delta E = E_{binding} - E_{hydration}$. When the ion enters the pore, it experiences an energy barrier of ΔE. When it leaves the pore, it experiences an energy barrier of $-\Delta E$. Therefore, the relationship between the ionic resistivity (R) and ΔE can be written as,

$$R \propto e^{-\Delta E/RT} + e^{\Delta E/RT} \qquad \text{(S1)}$$

Then ionic flux (J) is related to ΔE by,

$$J \propto 1/(e^{-\Delta E/RT} + e^{\Delta E/RT})$$

The ionic conductivity can be written as,

$$G \propto zJ \propto z/(e^{-\Delta E/RT} + e^{\Delta E/RT})$$

Considering $|\Delta E| >> RT$, we have

$$\ln(G/z) \propto e^{-|\Delta E|/RT} \qquad \text{(S2)}$$

Here z is the valence number. Clearly, G increases with the decrease of the absolute value of ΔE. This means when the hydration energy is approximately equal to the binding energy, the ionic conductance is maximum. We therefore summarized $|\Delta E|$ for all tested ions and compared them with the conductivity normalized by valence (Supplementary Fig. 16). The results indeed follow Eq. S2, except for Al^{3+}. For Al^{3+}, the ionic conductance is close to the leak conductance, and might not represent the "true" ion transport behavior of Al^{3+}.

These results suggest that the transport mechanism is governed by the competition between the energy required to shedding away the hydration shell and the binding energy provided by the macrocycles.

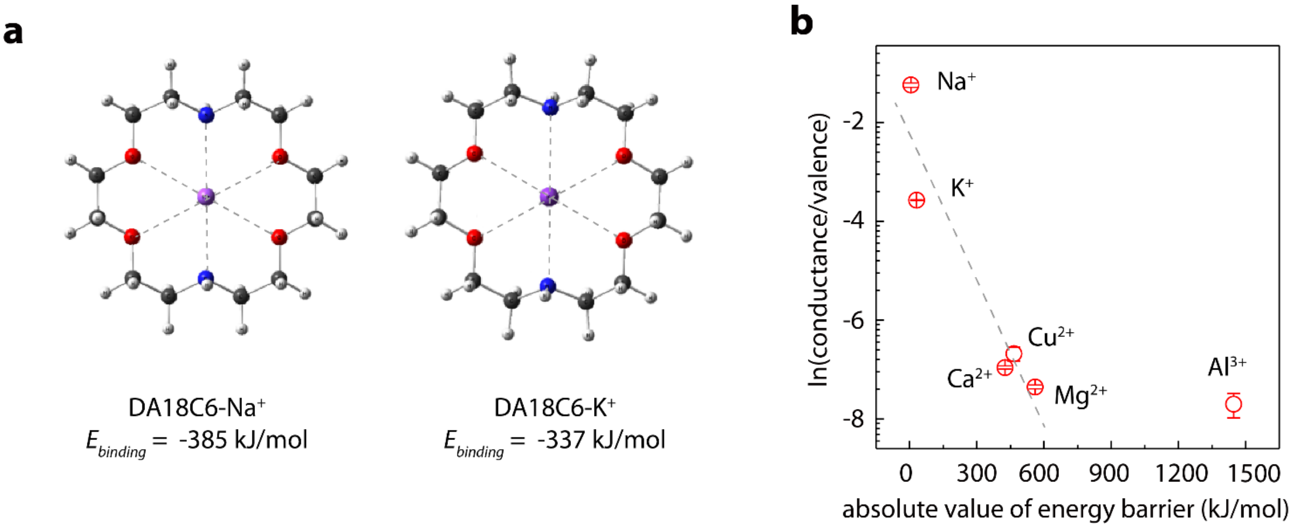

Supplementary Figure 16 | Transport mechanism analysis (a) The binding of DA18C6-Na^+ and DA18C6-K^+ computed by density functional theory. The binding energies of metal ion with DA18C6 are indicated. The optimized structures show that all metal ions will stay in the cavity of DA18C6 via interacting with O and/or N atoms. (b) For all tested ions except Al^{3+}, The logarithm of conductance normalized by valence is approximately proportional to the absolute value of energy barrier ($|\Delta E|$), in accordance with Eq. S2. Dashed line is eye-guide. For Al^{3+}, the ionic conductance is close to the leak conductance, and might not be trusted. These results suggest that the transport mechanism, and thereby the ion selectivity mechanism, are governed by the competition between the energy required to shedding away the hydration shell and the binding energy provided by the macrocycles. Error bars in all cases indicate the standard deviation of the data.

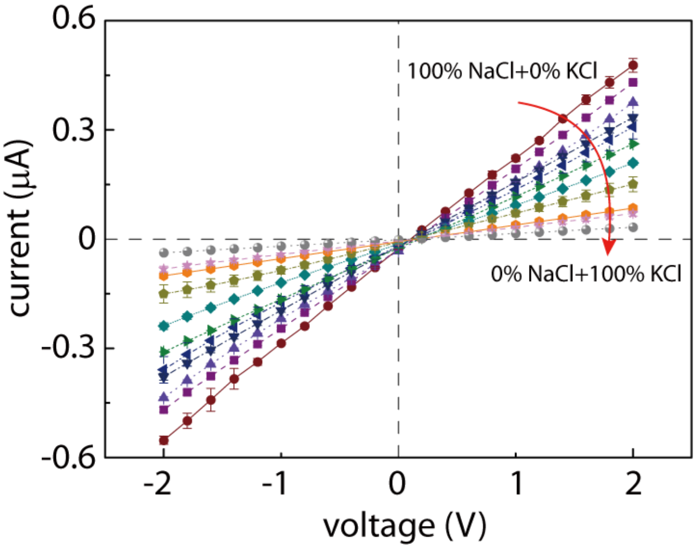

Supplementary Figure 17 | *I-V* curves of 0.1 M NaCl/KCl electrolyte wit variable Na^+ molar ratio. The Na^+ molar ratio was decreased from 100% to 0% with a 10% step. The conductance of the mixture decreased gradually with the decrease of Na^+ concentration. Error bars in all cases indicate the standard deviation of the data.

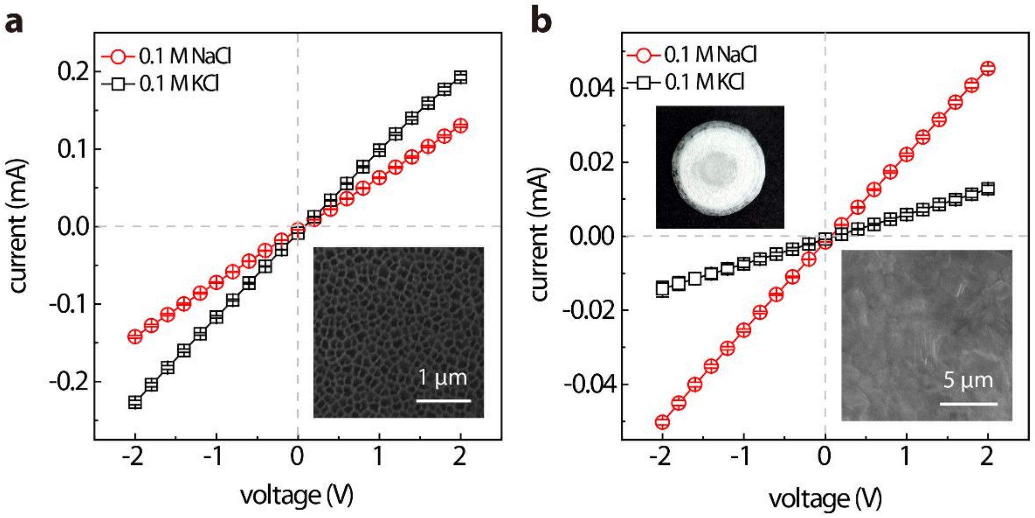

Supplementary Figure 18 | Na^+/K^+ selectivity of an AAO film (a) and DA18C6-nitrate/zinc hydroxide nitrate filtrated on an AAO film (b), tested with 0.1 M NaCl or 0.1 M KCl. Lower insets are corresponding top-view SEM images. Upper inset in (b) is a top-view optical image. Error bars in all cases indicate the standard deviation of the data.

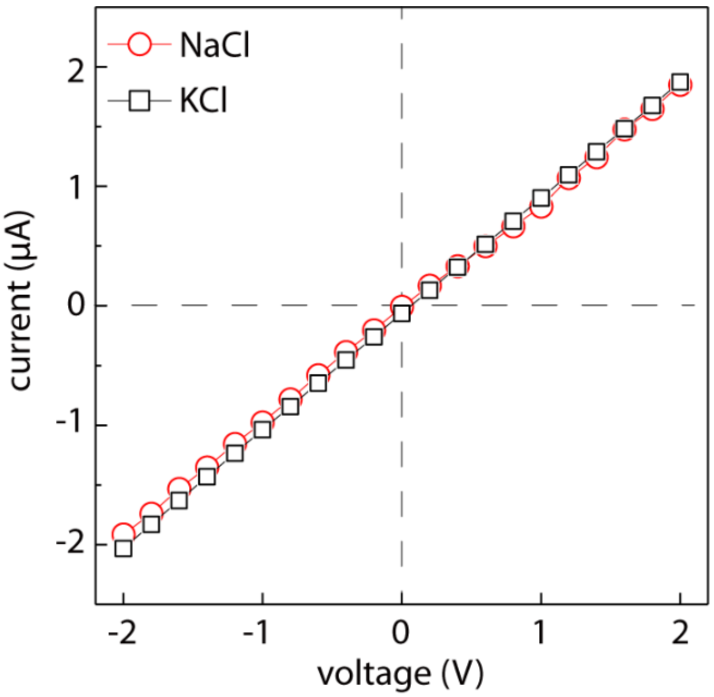

Supplementary Figure 19 | *I-V* curves of 0.1 M NaCl and KCl with blank micropipette, showing negligible selectivity.

Supplementary Table 1 | Crystal data and structure refinement.

Compound	$[C_{12}H_{28}N_2O_4](NO_3)_2$	
Empirical formula	$C_{12}H_{28}N_4O_{10}$	
Formula weight	388.38	
Temperature	296(2) K	
Wavelength	71.073 pm	
Crystal system	Monoclinic	
Space group	P 21/n	
Unit cell dimensions	a = 779.02(16) pm	α= 90°
	b = 1062.9(2) pm	β= 106.454(4)°
	c = 1181.2(2) pm	γ= 90°
Volume	0.9380(3) nm^3	
Z	2	
Density (calculated)	1.375 Mg/m^3	
Absorption coefficient	0.119 mm^{-1}	
F(000)	416	
Crystal size	0.360 × 0.290 × 0.250 mm^3	
Theta range for data collection	2.628 to 25.995°	
Index ranges	-9<=h<=9, -12<=k<=13, -10<=l<=14	
Reflections collected	7269	
Independent reflections	1835 [R(int) = 0.0258]	
Completeness to theta = 25.242Å	99.5 %	
Absorption correction	Semi-empirical from equivalents	
Max. and min. transmission	0.979 and 0.967	
Refinement method	Full-matrix least-squares on F^2	
Data / restraints / parameters	1835 / 0 / 118	
Goodness-of-fit on F^2	1.073	
Final R indices [I>2sigma(I)]	R1 = 0.0471, wR2 = 0.1446	
R indices (all data)	R1 = 0.0511, wR2 = 0.1482	
Extinction coefficient	n/a	
Largest diff. peak and hole	0.360 and -0.341 $eÅ^{-3}$	

Supplementary Table 2 | Atomic coordinates (×10[4]) and equivalent isotropic displacement parameters ($pm^2 \times 10^{-1}$). U(eq) is defined as one third of the trace of the orthogonalized U^{ij} tensor.

	x	y	z	U(eq)
O(1)	2196(2)	7120(1)	4483(1)	43(1)
O(2)	5854(2)	3490(1)	3093(1)	43(1)
O(3)	1361(2)	8721(2)	9493(2)	98(1)
O(4)	2775(2)	7776(1)	11052(1)	68(1)
O(5)	1649(4)	6704(2)	9501(2)	105(1)
N(1)	3121(2)	5231(1)	3091(1)	38(1)
N(2)	1881(2)	7710(1)	9971(1)	48(1)
C(1)	1942(3)	7695(2)	5513(2)	49(1)
C(2)	935(2)	6174(2)	3986(2)	46(1)
C(3)	1287(2)	5759(2)	2857(1)	44(1)
C(4)	3507(3)	4773(2)	1995(1)	46(1)
C(5)	5448(3)	4446(2)	2229(2)	47(1)
C(6)	7692(2)	3156(2)	3433(2)	49(1)

Supplementary Table 3 | Binding energy of DA18C6-M (M=metal cation) calculated by density functional theory, hydration energy of metal cations[1], and the deduced energy barrier (absolute value) of the ion transport.

Cation	Hydration energy[1] (kJ/mol)	Binding energy of DA18C6-M (kJ/mol)	Energy barrier (absolute value, kJ/mol)
Na^+	379	385	6
K^+	306	337	31
Ca^{2+}	1514	1048	466
Cu^{2+}	2030	1604	426
Mg^{2+}	1850	1290	560
Al^{3+}	4533	3086	1447

1. Gomer, R., & Tryson, G. (1977). An experimental determination of absolute half-cell emf's and single ion free energies of solvation. *The Journal of Chemical Physics*, *66*(10), 4413-4424.
